# Differences in clinicopathologic features and subtype distribution of invasive breast cancer between women older and younger than 40 years

**DOI:** 10.20407/fmj.2019-001

**Published:** 2019-09-25

**Authors:** Kaori Ushimado, Naomi Kobayashi, Masahiro Hikichi, Tetsuya Tsukamoto, Makoto Kuroda, Toshiaki Utsumi

**Affiliations:** 1 Department of Breast Surgery, Fujita Health University, School of Medicine, Toyoake, Aichi, Japan; 2 Department of Diagnostic Pathology, Fujita Health University, School of Medicine, Toyoake, Aichi, Japan

**Keywords:** Breast cancer, Young woman, Clinicopathologic characteristics, Subtype

## Abstract

**Objectives:**

We investigated and compared clinicopathologic features and subtype distribution of invasive breast cancer among women <40 and ≥40 years of age.

**Methods:**

We retrospectively compared clinicopathologic characteristics and subtype distribution of invasive breast cancer in women <40 and ≥40 years of age, in a cohort of 1,130 patients. Subtypes included luminal A (positive for hormone receptors [HR]—estrogen receptor [ER] and/or progesterone receptor [PR]—and negative for human epidermal growth factor receptor 2 [HER2] with low Ki67), luminal B (HER2^–^) (HR^+^/HER2^–^/Ki67^High^), luminal B (HER2^+^) (HR^+^/HER2^+^), HER2-overexpressing (HR^–^/HER2^+^), and triple negative (ER^–^/PR^–^/HER2^–^).

**Results:**

Breast cancers in younger women had unfavorable clinicopathologic characteristics, including larger tumors and more frequent node involvement. Subtypes among the 1,130 tumors were luminal A: 36.4%, luminal B (HER2^–^): 35.0%, luminal B (HER2^+^): 7.5%, HER2-overexpressing: 7.1%, and triple negative: 14.0%. The age groups significantly differed in subtype distribution (*P*<0.001). Luminal A subtype was more common in the older group (38.5%) than the younger group (16.2%), and luminal B (HER2^–^) was more common in the younger group (52.2%) than in the older group (33.2%; *P*<0.001).

**Conclusions:**

Breast cancers in women younger than 40 years have unfavorable clinicopathologic characteristics and are more likely to be luminal B (HER2^–^) and less likely to be luminal A than breast cancers in older women.

## Introduction

Breast cancer (BC) is the most common cause of malignancy-associated death for women in many countries.^1^ Although Japanese women have a lower incidence of BC than Western women,^2^ it has been increasing in Japan.^3^ Women younger than 40 years of age have a lower BC incidence than older women, but their BC incidence has been increasing.^3^

Moreover, BC in younger women has been shown to have worse prognosis,^4,5^ although not all reports bear this out.^6–8^ In general, tumors in younger patients are larger, are more likely to have more lymph node involvement, and are less likely to have favorable pathologic factors than those in older patients.^9–11^ Although younger age by itself has been suggested as a risk factor, some studies indicate that age alone is not a poor prognostic factor after adjusting for clinicopathologic factors.^12,13^

Recently, microarray-based technology has provided new genetic approaches for investigating complex clinical issues regarding BC outcomes.^14,15^ Remarkably, microarray studies have shown that BC is a heterogeneous collection of different subtypes characterized by distinct aberrations at the molecular level. Based on gene expression studies, BC can be classified into at least five distinct subtypes: luminal A, luminal B, human epidermal growth factor receptor 2 (HER2) overexpressing, basal-like, and normal breast. Differences in gene expression patterns have been associated with differences in clinical outcomes.^15^ In general, the luminal A subtype is associated with favorable outcomes whereas basal-like and HER2-overexpressing subtypes have poor prognoses.^14^

Protein expression has been shown to act as a surrogate for the tumor genomic profile when classifying BC into subtypes with distinct clinical outcomes and biologic characteristics.^16,17^ Recently, subtype classification by protein expression rather than molecular expression has become widely used because of its greater convenience. The St. Gallen consensus statement classifies BC subtypes by immunohistochemistry findings for estrogen receptor (ER), progesterone receptor (PR) (together, the hormone receptors [HR]), HER2, and Ki67 expression,^18,19^ into five major subtypes—luminal A (HR^+^/HER2^–^/Ki67^Low^), luminal B (HER2^–^) (HR^+^/HER2^–^/Ki67^High^), luminal B (HER2^+^) (HR^+^/HER2^+^), HER2-overexpressing (HR^–^/HER2^+^), and triple negative (ER^–^/PR^–^/HER2^–^)—which we used in this study.

The relationship between BC subtype and age is not well understood.^10,12,20–23^ We therefore compared clinicopathologic characteristics and subtype distribution of invasive BC between women older and younger than 40 years.

## Patients and methods

### Subjects

Between 2003 and 2014, 1,704 patients with BC were treated at Fujita Health University Hospital. This study excluded men, patients with stage IV, occult or noninvasive cancer, or bilateral disease, and patients lost to follow-up immediately after surgery. A total of 1,130 women with invasive BC were included. Patients were divided into two groups: younger women (<40 years of age) and older women (≥40 years of age). Histologic grades were assessed according to the Bloom and Richardson classification system.^24^ We investigated the relationship between clinicopathological factors (stage, T stage, pathological node status, histological grade, PR status, subtype distribution, chemotherapy, endocrine therapy, and types of operation) and the two age groups. This retrospective study was approved by the Ethics Committee of Fujita Health University (No. HM16-138).

### Immunohistochemistry

Immunohistochemical methods were described previously.^25^ Although surgical specimens were used as sample sources, core biopsies before neoadjuvant therapy were used for patients who underwent neoadjuvant therapy. Immunohistochemical staining was carried out using the SP1 and 1E2 (Ventana Medical, Tucson, AZ, USA) staining systems for ER and PR, respectively. Positive ER or PR status was defined as the presence of ≥1% positive cancer cells. Immunohistochemical assays for HER2 were performed using the Pathway anti-HER2/neu test (Ventana Medical). Fluorescence in situ hybridization (FISH) was performed using the PathVysion *HER-2* DNA probe kit (Abbott France SAS, Rungis, France). An immunohistochemistry score of 3+ or FISH amplification was defined as positive. Ki67 staining was performed using the monoclonal antibody MIB-1 (Dako, Glostrup, Denmark). The Ki67 labeling index was categorized as low (<14%) or high (≥14%).^26^ All markers were assessed with blinding to the clinical data.

### Breast cancer subtype classification

Tumors were classified into five subtypes based on the status of ER, PR, Ki67, and HER2 immunohistochemistry results: luminal A (HR^+^/HER2^–^/Ki67^Low^), luminal B (HER2^–^) (HR^+^/HER2^–^/Ki67^High^), luminal B (HER2^+^) (HR^+^/HER2^+^), HER2-overexpressing (HR^–^/HER2^+^), and triple negative (ER^–^/PR^–^/HER2^–^).

### Distant disease-free and overall survival by age group

Distant disease-free survival (DDFS) was defined as first distant recurrence or death from any cause. DDFS was calculated from the date of diagnosis to the date of distant recurrence or death. Overall survival (OS) was calculated from the date of diagnosis to the date of death from any cause.^27^ We assessed DDFS and OS in the two age groups.

### Statistical analysis

Statistical analysis was performed using SPSS 22.0 software (IBM Corp., Armonk, NY, USA). The chi-square test was used for contingency table analysis. Survival curves were generated using the Kaplan–Meier method.^28^ Survival comparisons were made using the log-rank test.

## Results

### Clinical characteristics of study patients

**Figure 1:**
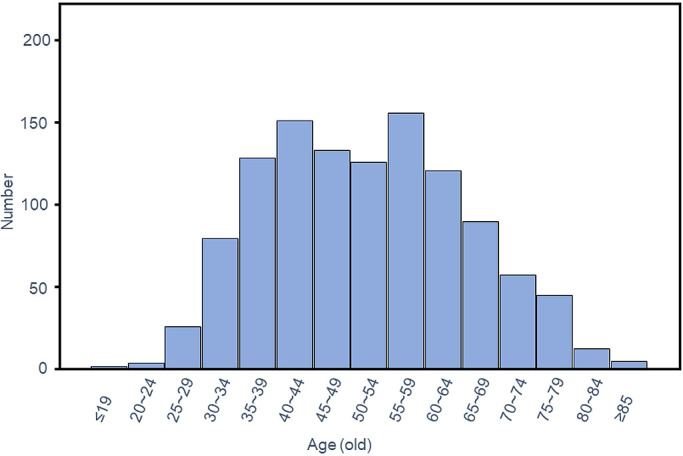
Distribution of age at diagnosis among 1,130 patients.

Distribution of age at diagnosis of the 1,130 patients is shown in Figure 1. Of the 1,130 patients, 111 (9.8%) were younger than 40 years and 1019 (90.2%) were older than 40 years. Table 1 shows their clinical profiles. Significantly more women in the older group had early-stage (T1) BC (49.8%) than did the younger women (37.8%; *P*=0.038).

Among the 1,130 patients, data on pathologic node status was missing for 36 patients, including two younger women and 34 older women. Of the two young women, one did not undergo axillary surgery. The remaining patient had no pathologic node involvement after neoadjuvant chemotherapy (NAC) and showed no evidence of negative lymph node status before NAC. Of the 34 older women with missing data, 28 patients did not undergo axillary surgery; no information regarding pathologic node status before NAC was available for six patients who underwent NAC. In total, 34 older patients had missing node status. A significantly higher percentage of the younger group (46.3%) had node involvement than did the older group (34.5%; *P*=0.032).

A significantly higher percentage of younger women had histologic grade 3 tumors (27.0%) than did the older women (15.2%; *P*=0.007). No data were available about for three women in the younger group and 30 in the older group.

### Biologic markers and immunohistochemical BC subtype

The two age groups did not significantly differ in HR or HER2 status. Interestingly, however, a significantly larger percentage of the younger group’s BCs were Ki67^High^ (79.3% vs. 57.3%, *P*<0.001).

Of the 1,130 tumors, 36.4% were luminal A, 35.0% were luminal B (HER2^–^), 7.5% were luminal B (HER2+), 7.1% were HER2-overexpressing, and 14.0% were triple negative. Their distribution by age group significantly differed (*P*<0.001; Table 2). Luminal A subtype was more common in the older group (38.5%) than the younger group (16.2%), whereas younger women were more likely to have luminal B (HER2^–^) than older women (52.2% vs. 33.2%).

### Treatment options

The two age groups did not significantly differ in percentages of patients treated with breast surgery or axillary surgery, or in rates of hormonal therapy or anti-HER2 therapy (Table 3). Chemotherapy was administered to 60.4% of the younger women and 44.7% of the older women (*P*=0.002).

### DDFS and OS by age group

**Figure 2:**
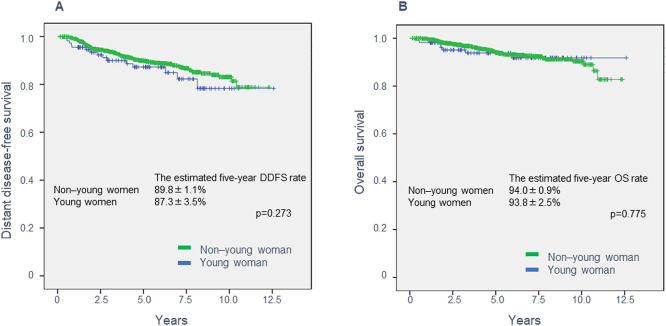
Distant disease-free and overall survival for 1,130 women with breast cancer. (A) Distant disease-free survival and (B) overall survival by age group.

Over an overall median follow-up of 5.10 years (range: 0.15–12.59 years), DDFS and OS did not significantly differ between the two age groups (Figure 2). The estimated five-year DDFS rate was 89.8±1.1% for BC in older women and 87.3±3.5% in younger women (*P*=0.273). The estimated five-year OS rate was 94.0±0.9% for older women and 93.8±2.5% for younger women (*P*=0.775).

## Discussion

The age-adjusted incidence rate of BC in Japanese women was reportedly 79.7 per 100,000 women per year in 2009.^29^ In the United States, it was 127.9 per 100,000 women per year in 2015.^30^ The peak age for BC is between 40 and 50 years in Asian countries but between 60 and 70 years in Western countries.^31^ In the United States, 6.6% of women with BC are diagnosed before the age of 40 in 2008 according to the Surveillance, Epidemiology and End Results database.^32^ In Japan, 7.7% of women with BC diagnosed between 2004 and 2009 were younger than 40 years of age according to Registration Committee of Japan Breast Cancer Society.^33^ The cut-off age for “younger” BC patients varies in different studies, although most investigations seem to use either the age of 35 or 40 years. In our study, 40 years was the cut-off age. The incidence of invasive BC in our younger group was 9.8%, which is higher than results from other reports.^32,33^ This may be because the distribution of age at diagnosis for BC differs between Japan and the United States,^31^ or because study participants had different background characteristics. Our study excluded patients with stage IV, occult or noninvasive cancer, and bilateral disease, but two previous studies included patients with all types of BC.^32,33^

BC in younger women reportedly has a worse outcome than in older women.^4,5^ However, this issue remains controversial. According to a population-based study in Switzerland, relative youthfulness did not affect survival.^8^ El-Saghir et al. also found that younger age does not have any adverse effects on survival in patients with BC.^6^ Moreover, a study by Chia et al. showed that younger women with BC have a better prognosis than older patients.^7^ A major reason for poor outcome in younger women is thought to be stage shift or aggressive phenotype. In this study, we examined clinical characteristics, subtypes, and clinical outcomes of a retrospective cohort of patients in two age groups. We found that breast tumors in younger women were larger, more frequently node-positive, and more frequently of higher histologic grade than those in older women. These findings are consistent with results from previous studies.^9–11^ Stage classification is based on the anatomical extent of cancer spread, and is a critical prognostic factor. Smaller tumors are hard to find in young patients because young women generally have dense breasts on mammography. Moreover, in Japan, mammography screening has been recommended biannually for women aged 40 years and over. It is a culturally accepted way to screen for BC that is covered by national health insurance. Our finding that older women had BCs diagnosed at earlier T stages compared with those in younger women could be partially explained by the mammography screening system for women aged 40 years and over. As the incidence of BC in women younger than 40 years is low and dense breasts make it more difficult to detect cancer using a mammogram, our results do not support the idea of lowering the age for routine mammography screening.

Conventionally, prediction of prognosis has been influenced by the anatomical extent of the tumor, reflected by stage classification, but tumor biology is apparently more relevant to prognosis than tumor size.^34^ Currently, BC is widely recognized as a heterogeneous group of different subtypes with varying clinicopathologic features and response to systemic therapies. Interestingly, we found that the luminal A subtype (usually associated with better prognosis than other subtypes) was more common among older women. By contrast, luminal B subtype, (usually an aggressive phenotype) was more common among younger women. These findings are consistent with the results of Partridge et al.,^23^ but differ from the results of Morrison et al.^20^ The distribution of BC subtypes also differs among different races.^35^ Variations in results among these studies might be caused in part by different sample sizes or different races. We don’t know why BC in young women was more likely to have high Ki67 expression, which is a marker for proliferation. This finding may be attributable to differences in plasma estradiol levels between the two age groups. Estradiol has been shown to enhance ER-induced proliferation of MCF-7 cells by stimulating expression of Ki67.^36^ As our older group includes postmenopausal women whose plasma estradiol levels are lower than those of premenopausal women, the younger women group might have higher Ki67 expression and a higher rate of luminal B subtype compared with the older group. Histological grade is decided by tubule formation, nuclear pleomorphism, and mitosis count. As proliferation and mitosis are related, BC in younger women might tend to have higher histologic grades than in older women.

Surprisingly, our results did not indicate any significant differences in DDFS or OS between the two age groups, even though tumors in the younger women were larger, more frequently had lymph node involvement, and were more likely to have unfavorable pathologic factors than those in the older women. Chemotherapy was used more frequently in the younger women than in the older women. Our data seems consistent with the finding that age is not a prognostic factor by van de Vijjver et al.^12^ and Ibrahim et al.^13^ The reason why there were no differences in outcomes between the two age groups in our study might be related to the small sample size. Our study might have lacked sufficient power to highlight the impact of outcomes. Other reasons might be differences in chemotherapy rates in the two age groups or relative shorter follow up time for outcomes.

Our study has some limitations. First, this was a retrospective study with data collected at a single institution. Accordingly, it includes biases related to all retrospective studies, such as selection bias. Second, the number of younger patients was small. Because relatively small studies might not yield definitive results, we must interpret the results with caution. A larger observational series might provide additional data. However, our study also contains several strengths. First, data on the two age groups were precisely collected at a single institution. Second, the relationship between BC subtype and age is now widely thought to be an important topic in the field of BC.

In conclusion, BC in women younger than 40 years have unfavorable clinicopathologic characteristics, and are more likely to be luminal B (HER2^–^), and less likely to be the luminal A than BCs in women older than 40 years. Further study with a larger number of patients is recommended to validate our findings.

## Figures and Tables

**Table1:** Breast tumor pathologic characteristics by age

Age group	<40 years *n*=111			≥40 years *n*=1019		*P*
	*n*	%		*n*	%	
T stage						
T1	42	37.8%		507	49.8%	
T2	62	55.9%		423	41.5%	
T3	3	2.7%		36	3.5%	
T4	4	3.6%		53	5.2%	0.038
Pathological node status						
Negative	57	51.4%		633	62.2%	
Positive	52	46.8%		352	34.5%	
Unknown	2	1.8%		34	3.3%	0.032
Stage						
I	39	35.1%		476	46.7%	
IIA	46	41.5%		331	32.5%	
IIB	18	16.2%		122	12.0%	
IIIA	3	2.7%		31	3.0%	
IIIB	4	3.6%		49	4.8%	
IIC	1	0.9%		10	1.0%	0.209
Histological grade						
1	21	18.9%		291	28.6%	
2	57	51.4%		543	53.3%	
3	30	27.0%		155	15.2%	
Unknown	3	2.7%		30	2.9%	0.007

**Table2:** Biological profiles and subtypes by age

Age group	< 40 years			≥40 years		*P*
	*n*	%		*n*	%	
ER						
Negative	28	25.2%		227	22.3%	
Positive	83	74.8%		792	77.7%	0.480
PR						
Negative	37	33.3%		348	34.2%	
Positive	74	66.7%		671	65.8%	0.863
HER2						
Negative	94	84.7%		872	85.6%	
Positive	17	15.3%		147	14.4%	0.801
Ki67						
Low (<14 %)	23	20.7%		435	42.7%	
High (≥14 %)	88	79.3%		584	57.3%	<0.001
Subtype						
Luminal A	18	16.2%		393	38.5%	
Luminal B (HER2^–^)	58	52.2%		338	33.2%	
Luminal B (HER2^+^)	10	9.0%		75	7.4%	
HER2 overexpressing	7	5.4%		73	7.2%	
Triple negative	18	16.2%		140	13.7%	<0.001

ER: estrogen receptor; HER2: human epidermal growth factor receptor 2; PR: progesterone receptor.

**Table3:** Treatment options by age

Age group	<40 years *n*=111			≥40 years *n*=1019		*P*
	*n*	%		*n*	%	
Breast surgery						
No breast surgery	1	0.9%		1	0.1%	
Breast-conserving surgery	64	57.7%		606	59.5%	
Mastectomy	46	41.4%		563	40.4%	0.155
Axillary surgery						
No axillary surgery	1	0.9%		28	2.7%	
ALND±SNB	47	42.3%		393	38.6%	
SNB	63	56.8%		598	58.7%	0.415
Adjuvant and/or neoadjuvant chemotherapy						
Not given	44	39.6%		563	55.3%	
Given	67	60.4%		456	44.7%	0.002
Adjuvant and/or neoadjuvant endocrine therapy						
Not given	29	26.1%		223	21.9%	
Given	82	73.9%		796	78.1%	0.308
Adjuvant and/or neoadjuvant anti-HER2 therapy						
Not given	96	86.5%		908	89.1%	
Given	15	13.5%		111	10.9%	0.405

ALND: axillary lymph node dissection; HER2: human epidermal growth factor receptor 2; SNB: sentinel lymph node biopsy.
